# Understanding the Role of Nature Engagement in Supporting Health and Wellbeing during COVID-19

**DOI:** 10.3390/ijerph19073908

**Published:** 2022-03-25

**Authors:** Patricia M. Darcy, Jennifer Taylor, Lorna Mackay, Naomi J. Ellis, Christopher J. Gidlow

**Affiliations:** 1 Centre for Health and Development, Staffordshire University, Stoke-on-Trent ST4 2DF, UK; n.j.ellis@staffs.ac.uk (N.J.E.); c.gidlow@staffs.ac.uk (C.J.G.); 2 School of Health, Science and Wellbeing, Staffordshire University, Stoke-on-Trent ST4 2DE, UK; jennifer.taylor@staffs.ac.uk (J.T.); lorna.mackay@outlook.com (L.M.)

**Keywords:** nature engagement, health inequalities, health and wellbeing, COVID-19, qualitative

## Abstract

The importance of natural environments in supporting health and wellbeing has been well evidenced in supporting positive mental and physical health outcomes, including during periods of crisis and stress. Given the disproportionate impacts of the COVID-19 pandemic have been greatest for those who are most vulnerable, understanding the role of natural environment and alternative forms of nature engagement in supporting health and wellbeing for vulnerable groups is important. This study explored how nature engagement supported health and wellbeing in those with a pre-existing health condition during the first UK lockdown. Semi-structured interviews were conducted with 17 adults with a pre-existing health condition and analysed using Interpretative Phenomenological Analysis (IPA). Four themes were identified: COVID-19 versus nature; Nature as an extension and replacement; Nature connectedness; and Therapeutic nature. The findings show the importance of nature in supporting health and wellbeing in those with a pre-existing health condition through engagement with private and public natural environments, micro-restorative opportunities, nature connection as an important pathway, and the therapeutic benefits of nature engagement. The present research extends the evidence-base beyond patterns of nature engagement to a deeper understanding of how those with existing health conditions perceived and interacted with nature in relation to their health and wellbeing during the first UK lockdown. Findings are discussed in relation to health supporting environments, micro-restorative opportunities, and policy implications.

## 1. Introduction

In recent years, there has been increased interest among policy makers, researchers and health professionals in the health and wellbeing benefits of natural environment engagement [1,2,3,4,5]. The importance of natural environments (green spaces, blue spaces, urban green spaces (UGS)) in supporting health and wellbeing has been well evidenced, through positive impacts on mental and physical health [6,7], psychological and physiological stress reduction [8,9], and restoration of cognitive processes [10,11].

Natural environments are thought to benefit health and wellbeing directly and indirectly. Nature exposure, including passive nature exposure (i.e., viewing or being in nature, but not engaging in an activity), quasi-passive nature exposure (i.e., exploring nature, such as touching and smelling plants) and active nature exposure (i.e., actively engaging with the natural environment), may lead to health benefits through improving affect [12,13] and cognitive functioning [14,15], facilitating recovery from stress [16,17], and enhancing well-being through the generation of social connectedness [18,19].

Much of the natural environment-health evidence relates to studies of proximal greenspace (i.e., public and private greenspace around the home), for which there is a significant body of evidence demonstrating the affective benefits [6,20,21]. Availability of private greenspace has been shown to have a greater influence than neighbourhood greenspace in relation to general health [22], and spending time in a garden is positively associated with health and wellbeing. Specifically, those who spend time in gardens are more likely to report better general health and life satisfaction, and reduced stress and BMI, compared with non-gardeners [23].

Modern living is also associated with increased time spent indoors and concurrent reduction in time and opportunities to engage with natural environments [24]. For populations who are older, or those with health problems or disabilities, engagement with the outdoors may be further impeded [25,26,27]. Such ‘vulnerable populations’ can spend up to 100% of their time indoors [28]. Nature-based indoor environments have previously been identified as a potentially effective health promoting intervention [29], where biophilic indoor environments can play a significant role in effecting health and cognitive function [30]. However, exploring alternative types of nature engagement is also required, where the associated positive health outcomes can be realised through easily accessible, safe, and cost-effective interventions [31].

In relation to vulnerable subpopulations, nature-based interventions have a good evidence base for positive mental health outcomes [32] and have the potential for a much wider impact across a range of population groups [33]. Within clinical settings, exposure to nature has been shown to reduce stress, anxiety, and pain through distraction [34,35,36], diverting patient’s attention to a pleasant environment, and in turn increasing positive affect and reducing negative affect [37].

### 1.1. Nature Engagement and COVID-19

During COVID-19, there has been an increase in use of public natural environments and a change in usage patterns [38,39,40]. More than 40% of those surveyed in the People and Nature survey (in England) reported that nature, wildlife, and visiting natural spaces have been more important to their wellbeing since the COVID-19-related restrictions began [39]. UGS closures as a responsive measure during COVID-19 in some places sparked criticism for compounding existing health inequalities [41]. While ensuring the provision of accessible outdoor natural spaces has an important role in mitigating health inequalities and in achieving sustainability (e.g., SDG 3: good health and wellbeing and SDG 11: sustainable and resilient cities) [42], alternative forms of nature engagement for those with worse access to outdoor natural environments (e.g., those with no access to a private or shared garden [43]) or who are less able to engage with them (e.g., due to chronic illness or disability) warrants further study.

Alternative forms of nature exposure may have greater relevance in crisis situations where people are required to stay at home during periods of lockdown and to ensure equitable health outcomes for all population groups [44,45,46]. Proximal nature, such as private gardens, small UGS, views of nature and bird sounds may provide micro-restorative opportunities through direct and instant interaction with nature and facilitate more effective opportunities for restoration compared to neighbourhood greenspace [47,48].

Given the restrictions on mobility and social interactions during COVID-19, there has been an increase in digital technology use [49]. Evidence further suggests an increase in digital nature engagement to support wellbeing during COVID-19, including webcams to view wildlife and natural environments [50], engagement with nature videos via social media [51], and online nature challenges [52,53]. Nature websites, such as the RSPB website, saw an increase in visits by 69% from the previous year, with 79% new users accessing the website [39]. The UK’s largest nature challenge ‘30 days wild’ reported increased sign-ups during the first UK lockdown, with twice as many care homes participating [53], with evidence of psychological benefits for up to 2 months afterwards [54].

### 1.2. Previous and Present Research

During the COVID-19 public health crisis, natural environments have been shown to have a valuable role in supporting health and wellbeing through a range of interactions: intentional interactions, defined as purposely being in or experiencing natural environments, e.g., spending time in the garden or gardening [55,56], or spending time in public greenspace [57,58]; incidental interactions, i.e., physically present in natural environments, where the interaction is unintended, e.g., walking to work and viewing street trees [59]; and indirect interactions, i.e., not physically present in natural environments, e.g., a nature view through a window [46,60].

Much of the research to date has involved cross-sectional surveys in the general population, exploring associations between exposures and outcomes at the same single time-point. It is important to understand how different types of nature engagement might elicit health and wellbeing benefits, particularly in vulnerable populations who have been most impacted by the COVID-19 pandemic and related restrictions [41], yet less able to realise the potential health benefits of natural environment engagement.

### 1.3. Aim and Scientific Contribution

The present research is the first qualitative exploration of how those with a pre-existing health condition engaged with nature during the first UK COVID-19 lockdown (23 March–18 June 2020). During this period, those considered high risk were strongly advised to stay at home or shield in accordance with UK government guidelines. By gaining insight into participants’ subjective experiences, the study aimed to extend the evidence-base beyond patterns of nature engagement to a deeper understanding of how those with existing health conditions perceived and interacted with nature in relation to their health and wellbeing. To this end, IPA was used to give a voice to those with lived experience, raising awareness of their experiences which can thus affect the ways in which policy is discussed [61]. By providing valuable insight and illumination of the subject through an exploration of participants’ lived experiences during a global pandemic, recommendations are made regarding the potential for nature engagement to support vulnerable groups, including during public health crisis.

## 2. Materials and Methods

The study took a phenomenological approach. A double hermeneutic process was applied in line with IPA, whereby the researchers aim to understand an experience from the participants’ perspectives through a process of interpretative activity applied to participants’ meaning making [62]. An idiographic approach is central to IPA, where the researcher aims to garner a deeper understanding of first-person experiences, and individual cases, before moving to general claims [63].

### 2.1. Participants and Recruitment

**Figure 1:**
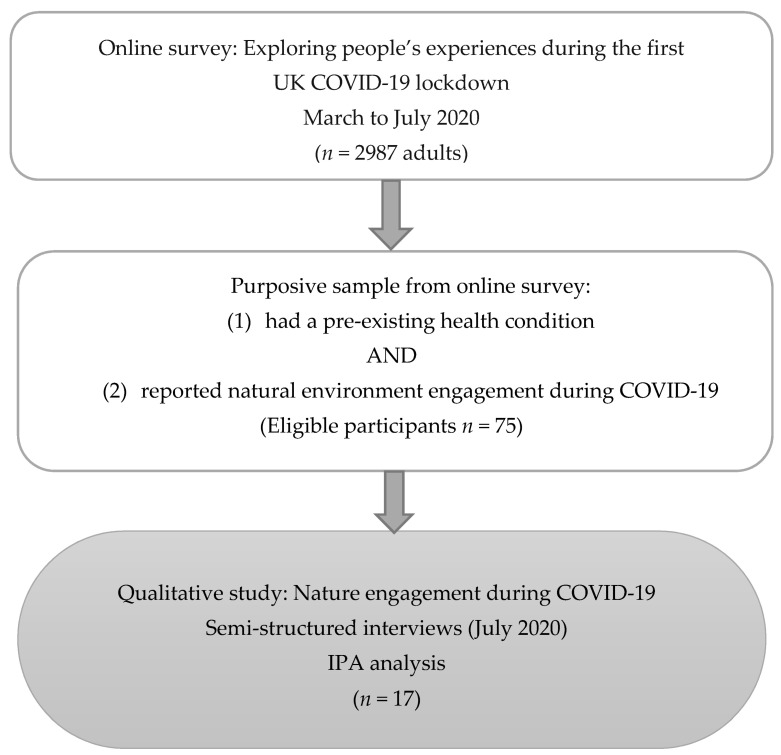
Study flow chart.

Participants were purposively sampled from a cohort of respondents to an online survey administered by the Centre for Health and Development (CHAD), Staffordshire University (March to July 2020). This initial online survey explored people’s experiences during the first COVID-19 lockdown (*n* = 2987 adults). The online survey comprised a range of data, including socio-demographics, health status, and responses to a range of quantitative and open-ended questions, including exploring engagement with nature (e.g., gardening, using natural environments) during the COVID-19 pandemic. All online survey respondents were asked to indicate their consent to be followed up for further research (Figure 1).

Participants for the present study were adults (aged 18+ years) residing in the UK who had a pre-existing health condition and self-reported using natural environments as a self-management activity during COVID-19. Participants were purposively sampled in line with recommendations for IPA [64]. IPA involves a detailed interpretative understanding of first-person experiences before general claims are made and therefore smaller sample sizes are typical of IPA to facilitate a rich and in-depth analysis [64]. While the sample size of the present study (*n* = 17) can be considered large for IPA [65], other researchers have highlighted no specific rules regarding IPA sample size [64]. A larger sample size was deemed beneficial here to explore divergences between cases to highlight the diversity of experiences in this sample.

An information sheet including a description of this study and a consent form were emailed to eligible survey respondents (*n* = 75), of whom 18 expressed an interest in taking part in a one-to-one interview (telephone/online), but one was later excluded (no pre-existing health condition).

Participants were aged from 28 to 75 years (females *n* = 12, males *n* = 5). Fifteen identified as having a pre-existing physical health condition and two participants identified a previous history of mental health difficulties (Table 1).

### 2.2. Patient and Public Involvement (PPI)

An established PPI group for those with a limiting chronic health condition informed the study design. An interview topic guide was developed, informed by responses to the open-ended survey questions regarding natural environment engagement and from PPI. The final topic guide was piloted and amended based on feedback from PPI. Topics included how and why people engaged with nature; perceived impacts; challenges in natural environment engagement during COVID-19; changes in perceptions and attitudes (Supplementary Tables S1 and S2).

### 2.3. Data Collection

Eighteen one-to-one semi-structured interviews were conducted in July 2020 by the first author (P.M.D.), 17 of which provided usable data and formed the final sample. Average interview duration was 50 min. All interviews were audio-recorded and transcribed verbatim for analysis, with field notes made by the interviewer during each interview. Participants received a GBP 10 online retail voucher in appreciation of their participation.

### 2.4. Data Analysis

All transcripts were entered into NVIVO software (Release 1.3, QSR International, Melbourne, Australia) and analysed following the six stages of IPA processes using an inductive and iterative approach [66]. This included familiarisation with data through immersion by reading and rereading the transcripts; creating initial notes through focusing on content, language, context, interpretative comments, and personal reflexivity; transforming initial interpretative comments and notes into themes; grouping themes into clusters by identifying patterns; generating a narrative account to include the participants account of their experience (which was treated as an idiographic case) and the interpretative account of the researcher.

In addition, guidance on conducting IPA processes with a research team was followed [67] as three authors (P.M.D., L.M., J.T.) were involved in the initial coding process. Before analysis began each team member engaged in an individual bracketing exercise, i.e., conducted a written reflection on the subject matter to identify their perspectives and biases. P.M.D. was undertaking a PhD on natural environments and health at the time the study was conducted, while L.M. and J.T. were unfamiliar with the research topic and related literature. The bracketing exercise and a team of coders was used in the present study to (a) deepen the research team’s understanding of the findings, and (b) to increase the credibility of the research through challenging the coding process i.e., to determine if coding was inductive, deductive from an existing theme or as a bias reflected in the bracketing document [67,68].

P.M.D., L.M. and J.T. coded the first transcript separately identifying themes. All team members discussed the emergent themes to identify and agree the superordinate themes. Following guidance for IPA [67], for subsequent transcripts (i.e., from participant 2 onwards), L.M. completed the coding of participants 2–5, P.M.D. participants 6–10, 12, 13, and J.T. participants 11, 14–17.

Each transcript was analysed using the themes from the first transcript to orientate analysis of subsequent transcripts. New themes were identified for each transcript, with convergences and divergences found. After each set of themes was generated, discussions with each individual coder were used to agree superordinate themes. Themes were then merged and reconceptualised by P.M.D. A final discussion was conducted with P.M.D., L.M., J.T., and N.J.E. to agree the definitive superordinate themes and subthemes and these were interpreted based on the research question.

## 3. Results

Four superordinate themes were developed which capture the role of nature engagement in supporting health and wellbeing during COVID-19: COVID-19 vs. nature; Nature as an extension and replacement; Nature connectedness; and Therapeutic nature (Table 2). These are summarised with illustrative quotations (using pseudonyms to protect participant identities). A figure summarising the results, illustrating how the superordinate themes and subthemes are connected and relate to each other is presented in Supplementary Material Figure S1.

While COVID-19 public health restrictions had an impact on the population generally, for those with a health vulnerability this impact often led to a greatly reduced world: “*My world was a very small place, my home, my garden, and walking distance from my house basically*” [Wendy]. Daily impacts included social isolation from friends, family, peers, colleagues, neighbours; a loss of independence and autonomy; recreational and work travel restrictions; changes to working environment and conditions; and impacts to mental health and wellbeing. Those who were shielding considered themselves to be “*in a slightly different position*”, where stricter guidelines resulted in being confined to one’s home and within a “*bubble*” [Florence]. For participants who were not shielding, but considered themselves to be vulnerable, some made the decision to keep their world restricted to their immediate home environment: “*we haven’t been outside the house, well outside the garden, outside the property at all*” [Hannah].

Fear associated with health vulnerablity was a deterrant for some participants in engaging with the world beyond the confines of their immediate environment. For those in shared living facilities, fear was also a motivator for remaining indoors and isolated away from others: “*I have found that a lot of people my age in the blocks, in the retirement block, some of the people still haven’t gone out. They don’t want to go out and they won’t go out until there is a vaccine*” [Stacey]. The impact of COVID-19 was therefore significant in shaping the lives of participants, particularly for those who were more vulnerable, where they continuously weighed up the risks of engaging with the outside world and adapted, reduced, or restricted their engagement with such spaces as a result.

### 3.1. Theme 1: COVID-19 versus Nature

This theme encapsulates the dichotomy of the COVID-19 pandemic versus nature and participants’ experiences of navigating this contrast. Despite living in a ‘reduced world’ in terms of the daily lived impacts of the pandemic, engaging with nature provided opportunities for participants to positively experience their world, albeit in a differentially constructed way than previously.

In contrast to the stress and uncertainty resulting from the pandemic, nature afforded a sense of constancy and normality, safety, control, reassurance, and escapism. In this way, nature engagement was integral in developing and supporting psychological resilience.

#### 3.1.1. Subtheme: Nature as an Escape from COVID-19

Benefits mainly focused around helping participants to cope, particularly through nature providing an escape, getting “*away from what was going on*” [Barbara]. Related to this, some participants spoke specifically about the benefits of natural environments providing respite from the constant media coverage of COVID-19. For Wendy, getting outdoors in nature provided an escape from both the negativity of the media content and the reality of the pandemic: “*In April when a lot of people were dying it just got a bit much. I felt that I had to ration myself, didn’t listen to the news when I woke up in the morning, we went out and had a walk instead.*” In this way, natural environments provided opportunities to ’shut oneself off from the world’, highlighting the shift in the meaning of this phrase previously associated with staying indoors:

Florence: “*it has helped with anxiety and stuff just being able to get outside and not listen to the news or not hear what is going on… it’s a different way of shutting yourself off from the world, being outside rather than being inside.*”

The concept of being present in one’s immediate environment was also referenced in relation to a sense of sanctuary private outdoor space provided. Private greenspace acted as a “*bubble*” helping participants to disconnect from a reality beyond the garden’s boundaries:

Katie: “*[my garden is] very important because otherwise, if I allow my mind to drift beyond the edges of my bubble… I feel quite anxious. But actually, here inside my bubble… I can pretend the world is OK.*”

Despite participants’ changed world, the cycles of nature continued, providing reassurance and hope, as expressed by Olive: “*So, it’s nice to see nature is still carrying on, it will still continue*”. However, the underlying tension of having to navigate the realities of COVID-19 remained evident. While nature facilitated an essential escape and reassurance in uncertain times, in other environments (e.g., online groups), it was sometimes challenging to exert the same level of control and sanctuary where conversations would steer towards COVID-19. A fear of inappropriateness due to the national mood, ‘*it wouldn’t have felt right*’ and a potential for ‘*backlash*’ also deterred William from sharing nature photos on social media.

#### 3.1.2. Subtheme: Adaptations and Considerations

Accessing public natural environments still needed to be adapted and considered because of the risks associated with COVID-19. Some participants chose to avoid local green and blue spaces due to fears related to their own health vulnerability and the risks posed “*because of the number of other people going there*” [Sally]. Instead, the safety afforded by private greenspace was prioritised whilst considering alternative ‘*safer*’ options in the future. Participants referred to how their awareness of COVID-19 and the risk of transmission was at the forefront of their mind when considering accessing natural environments and, for some, this risk outweighed the benefits of using these spaces:

Andy: “*During COVID-19, we have never seen so many people walking on the public footpath… the increased numbers of people… made us ironically utilise that open land less…Because you were very aware that people were touching gates, people were, you know, in close proximity, the paths were quite narrow, so you were going have to somehow cross people.*”

For Lori, accessing her local UGS became unmanageable, while her private allotment space was not amenable to child’s play, resulting in a loss of freedom for her and her daughter:

Lori: “*we couldn’t even go to our local park area just because of how many people were suddenly using it, so I think we tried a couple of times but after one or two visits it just became clear that it just wasn’t workable, particularly with a small child… I found it quite difficult to adjust to.*”

However, several participants also made active choices in engaging with public natural environments. Strategies were devised to facilitate safer use, such as going at quieter times, accessing places with booking systems, bringing hand-sanitiser, choosing natural environments with wide paths, or accessing quasi-natural environments that were considered safer (e.g., garden centres).

While engaging with public natural environments was not without its challenges, accessing local green and blue spaces provided a sense of purpose (e.g., dog walking) and ‘normality’ through engaging in an unrestricted way similar to pre-COVID times. The possibility of experiencing more normal social interactions and the ability to meet others in a safe and socially distanced way, once restrictions eased, were integral to engaging with these spaces. However, accessing public natural environments after shielding or a confined period at home could initially be a daunting experience:

Katie: “*I think having somebody with me was reassuring as well because it is quite anxiety provoking when you have not been anywhere for 3 months to suddenly be told you can just do whatever you want…. I haven’t got the safety of knowing that I am close to home and nobody else will be there.*”

#### 3.1.3. Subtheme: Safety and Control

Participants identified a key difference between private and public outdoor spaces, in feeling safe and protected. There was a strong sense of personal ownership of private outdoor space which generated a sense of control, security, and separated participants’ personal world from the world of COVID-19:

Andy: “*COVID-19 can make you feel that you have no control, well let’s face it we don’t do we? That you have no control over your world, that the world is going mad, there’s mayhem and death out there, and when you are in the garden it gives you the sense that you can control your environment, your immediate environment.*”

Although being outside and engaging with public natural environments was important for some, participants’ efforts to navigate the outside world in the context of their health vulnerability and COVID-19 is particularly apparent when considering participants’ preferences for private green space:

Kevin: “*COVID-19 in a way has frightened me because I don’t want to go out, I don’t want to be in, I feel very vulnerable… So having that private [outdoor] space where I can control who comes and who doesn’t is absolutely one hundred percent essential.*”

The importance of feeling safe and weighing up the risks was also reflected by Lori who had a private allotment. In this instance, her allotment became a physical “safe space” by the physical boundaries that separated her allotment from others, and the control she could exert over that space. In a similar way, the allotment facilitated a psychological safe space: “*we don’t own it, but it is our space… I think it became a safe space because it was somewhere that I could go and just get on with things and kind of block stuff out a bit*.”

For participants who were shielding and aware of their vulnerability, private greenspace also served as a “*safe space*” for exercising. In this way, private greenspace acted as an important health supporting environment and reinforced participants’ motivation and intentions to maintain physical exercise.

### 3.2. Theme 2: Nature as an Extension and Replacement

Building on participants’ experience of a reduced world due to the impacts of COVID-19, nature became an important extension and replacement to the places, spaces, and activities participants would have engaged with pre-COVID. This included engaging with private greenspaces (private domestic garden, backyard, small holding, private allotment), public natural environments (green and blue spaces), and micro-restorative nature experiences (digital nature engagement, nature views, artistic nature, and indoor nature). Additional types of nature engagement, such as subscribing to a nature journal, watching gardening programmes or nature documentaries were also identified. This importance of seeking out nature in any form, was summarised by Wendy: “*if I don’t have access to nature… I will still try to find the access to nature either through my house plants or through looking at pictures or whatever, just a very fundamental thing*.”

#### 3.2.1. Subtheme: The Garden as a Passive and Active Space

The private domestic garden took on new meaning and significance for participants during COVID-19, becoming an important extension and providing valuable space as ‘another room’: “*[the garden] became a part of the house, it was like an extension… The patio doors were always open*” [William].

While this blurring of boundary between indoor and outdoor space was echoed by many, conversely for those working from home, the garden also acted as a boundary between work and home life. The use of the garden space for breaks throughout the day, and switching off after the working day, supported participants in managing homeworking. Outside of the working space, the garden as an outdoor room was an important health and recreational space for those restricted to their home environment. Access to fresh air and sunshine in the garden were significant in supporting wellbeing for many, especially those shielding, while also facilitating opportunities for dining and relaxing outside:

Florence: “*if you have been stuck for 18 weeks like we have been, just being able to go out and sit in your garden, [it] makes you feel happy. A lot happier sitting outside than sitting inside.*”

For Florence who was shielding, personal, and familial benefits were intertwined in using the garden, providing valuable psychological space and time for self, while her children used it as a space for play. In contrast, Susan described returning to the family home during COVID-19, as the garden providing a valuable opportunity to claim personal time and space for herself away from the family unit. “*Listening to music*” outside gave her a connection to a “*festival*” and an opportunity to think about her future.

Where lockdown restrictions limited social interactions the garden acted as an important replacement for social contact with family members, especially for those who were considered high risk. In this way the garden supported social interactions in a safe way. The clement weather during the first lockdown also facilitated more time in the garden, and a greater use of the garden as an extension to the home environment. Some participants moved from an exclusively active to a greater passive use of the space. Sitting in the garden was also an opportunity to plan, but for others being in the garden, appreciating nature and enjoying the rewards of their gardening was highly valued:

Philip: “*it was the first time I think for years that we just sat in the garden, you know and just done nothing.*”

Being restricted to their home environment due to health vulnerability also gave participants an opportunity to undertake something different. This included using the garden for exercising, growing new things, landscaping, and completing outstanding projects. For most, this also served as an important way of coping by providing a sense of purpose and enjoyment:

Hannah: “*I said look we are going to be here for the whole of the growing year, so I can grow lots of different things, so we looked on it as more of a positive opportunity to use that and be out there every day, looking after things and tending them.*”

As reflected by Hannah, gardening provided an important structure. For some, there were intrinsic rewards for their efforts, such as cultivating a sense of pride from their achievements and receiving positive appraisal through the sharing of garden produce, creative activities, and successes. For others, however, empty shelves, concerns over food security, and fear associated with accessing supermarkets acted as motivators for growing their own. In this way, private garden space afforded a sense of control and a way to manage participants’ fears and uncertainties associated with the pandemic.

Private outdoor space also provided opportunities for physical exercise and play for families who were shielding, replacing opportunities normally available. For others, gardening became an indirect means of physical activity, replacing other more intentional forms of exercise, resulting in both cognitive and physical benefits to health and wellbeing:

Andy: “*I actually lost that half a stone in the first four weeks. So, a major benefit for me was actually getting back to my ideal weight and I wouldn’t have done that if it hadn’t been for COVID-19.*”

For Philip, however, despite experiencing physical difficulties due to his health, it was not the physical, but the cognitive benefits that served as a key motivational factor for him in pursuing gardening activities: “*I like the problem solving*”.

#### 3.2.2. Public Natural Spaces as an Extension

In contrast to private greenspace, local green and blue spaces facilitated a greater sense of psychological and physical space, and an enhanced sense of being away, escape and switching off:

Susan: “*I like the fact now that I can actually escape the house without being like mollycoddled all the time, I can just go and like sit somewhere on my own.*”

Here, Susan suggests a sense of restriction beyond those attributed to COVID-19, where others may have been concerned about her vulnerability. In this way, local natural spaces, where accessed, acted as an important extension beyond the home environment:

Florence: “*even being outside in the garden, you can still feel a little bit trapped and a little bit claustrophobic but being out on the nature reserve it’s much more of a release because the space is bigger. You don’t feel as trapped.*”

As reflected by Florence and others, public natural environments offered a greater diversity of sensory and nature experiences over and beyond that of private greenspace. Such biodiverse spaces were a valuable resource for psychological stimulation and enrichment and facilitated a greater sense of ‘being away’. For William, who had a private graveled garden, accessing local greenspace during lockdown was particularly significant as “*it broke up the day… gave us something to look forward to*” and provided a change from the daily home routine, where work, home-schooling and family life operated.

This reflects the importance of local natural spaces as an important extension in participants’ lives, where limitations were identified with private or shared garden space. Most participants were unable to access natural environments further afield, due to lockdown associated travel restrictions, or concerns about health vulnerability in using public transport, and/or accessing natural spaces with greater numbers of people. Participants spoke about their hopes and intentions to visit these spaces at a future point in time, after restrictions had eased or when they felt it was safe enough for them to do so. For some, digital nature engagement became an alternative way to ‘access’ these types of natural environments safely in the interim period.

#### 3.2.3. Micro-Restorative Nature

Digital nature engagement, window views, artistic nature and indoor plants provided participants with various opportunities to connect with nature, while simultaneously affording an escape from the immediacy of indoor environments and a respite from the wider COVID-19 pandemic.

Throughout lockdown, digital nature was commonly used by participants as a replacement, particularly to view locations and natural environments people missed, e.g., blue spaces. The internet was increasingly used as a resource for educational purposes, extending participants’ knowledge of wildlife and gardening. While most reported an increased use and engagement with digital nature, differences in experiences between real and digital nature were simultaneously noted. This was reflected by Karen who drew comparisons between both types of nature engagement:

Karen: “*The online [nature engagement] is much more social because I am interacting with people, whereas there is much more solitude whether I am out in my own garden or whether I am just walking out in the countryside. The purpose when I am outside, was not to meet people, it was to see and breathe the open air and the countryside.*”

Viewing familiar natural environments online (i.e., YouTube, webcams, Instagram, National Trust), evoked mixed feelings. For some, online videos, and webcams of natural environments (specifically blue spaces) brought optimism, hope, and nostalgia:

Hannah: “*I like to see it [the beach] and places that we have been, so if you can see it, and imagine being there, and thinking, when this is over, we shall be there again.*”

For others, viewing such places resulted in ambiguous feelings, expressed by Andy as optimism followed by sadness and uncertainty about when that might be possible again: “*I had mixed feelings about it, because sometimes you would say isn’t it lovely to see that garden and then I feel these waves of depression come over me, if I can’t go there.*” It was acknowledged that digital nature often lacked a sensory experience, evoking frustration for some. Nonetheless, digital nature provided valuable opportunities to connect safely with natural places that were important to participants, extending their worlds beyond that of their immediate environment.

In addition to digital nature, being able to view nature from the indoor environment was an important consideration during lockdown, especially for those working from home. A workspace that had a view of nature and daylight was considered critical, providing a sense of normality and reassurance:

Barbara: “*it sort of gives you a couple of minutes every hour to just sort of look at, repeat and you think ‘right yes, it’s not too bad’. Rather than being stuck in the solid four walls.*”

The language referenced by participants demonstrates a strength of feeling of release and escapism, which often contrasted to feelings of ‘being stuck’ or ‘trapped’ due to the restrictions associated with COVID-19. Similarly, for others, a view of nature from indoors facilitated an extension and escape beyond the immediacy of the interior home environment, captivating and holding attention:

Philip: “*I am looking out at the scene and it’s like at the moment there’s so many shades of green, I am looking at this one tree, and when the wind blows it’s like a thousand windchimes silently in the breeze and could sit and watch it for hours, in fact I do.*”

For others, the lockdown period provided valuable opportunities to engage with nature in an artistic form, as either a new activity or a re-engagement with a previous creative interest. Engaging creatively with nature afforded opportunities to develop a new skill, provided a sense of achievement and social benefits, was a means of expressing beauty, and an enjoyable and fun activity. For some, this was sparked by an increased awareness of nature during the pandemic coinciding with more time and opportunity to engage with this activity:

Wendy: “*I started drawing again so I look back and I think well I started off life with nature and drawing and here I was back at this historic point in time under completely different circumstances doing the same thing.*”

Here, Wendy hints at a nostalgic element, where the pandemic provided her with time to reconnect with both nature and creativity, activities she would have engaged in her childhood years. Nostalgia was also expressed by Lori; through re-connecting with a childhood activity through nature play with her daughter:

Lori: “*going out and collecting, you know, leaves and twigs and stuff like that and making a picture out of it. You know, the sort of stuff that you don’t really tend to do in your 30s but is a bit more socially acceptable and you know, to do with a 3-year-old but it’s actually still quite fun even if you are in your 30s.*”

For most, private greenspace took precedence over indoor nature; however, some had access to a greenhouse or polytunnel and used it to propagate plants, while others started off seedlings or grew herbs indoors. Working from home also provided more time and opportunity to tend to and enjoy indoor plants, which previously might have been a chore. In this way, nurturing nature indoors also provided a sense of purpose and positive reward: “*I am like the proud mum watching them [cacti seeds] growing*” [Rachel] and brought added value as a ‘green space’ within the domestic house.

### 3.3. Theme 3: Nature Connectedness

Nature connectedness was important in supporting participants’ health and wellbeing through sharing, connecting with, and noticing nature. Sharing nature included sharing plants and garden produce; sharing nature conversations, nature photos and videos; all which supported social engagement during a time of increased isolation. Connecting with nature was related to connecting with self, others, and with personal, work, and cultural identities. Noticing nature facilitated a connection to nature, through increased available time and opportunities. Noticing nature and acknowledging the role it played in supporting participants’ health and wellbeing in turn led to a greater appreciation of nature and a change in pro-environmental and pro-social attitudes and behaviours.

#### 3.3.1. Subtheme: Sharing Nature

Sharing nature was one way of managing the impact of COVID-19 on participants’ lives. The extension of participants’ world through nature was linked to a sense of community, interconnectedness, and conscientiousness. Digital technologies acted as an important medium in facilitating this extension (see nature as an extension and replacement theme), through supporting social interactions at a time when there were reduced opportunities to engage with others and visit places normally accessible. For some, staying connected with family members involved learning or teaching others to use new technology:

Sally: “*The hardest bit really was trying to train my parents in using things like WhatsApp so that we could share the garden with them.*”

Social media groups encouraged a sense of community through the sharing of gardening activities and advice. For some, joining likeminded nature groups was a new activity which brought friendships. For Jon, sharing photos of familiar natural environments with others online, forged a “*real sort of connection… it feels like a community*”. This sense of interconnectedness and community was further reflected by Andy in his “*sharing the good [produce] of today*”, and Rachel who “*passed them [plants] on [to friends]*”. Sharing experiences and the importance of sustaining community and connection was present throughout; in this instance Olive opened-up her small private garden space in her shared living community:

Olive: “*I say to people ‘do you want to sit in the area of the garden, you are welcome to. Even if you just want to sit in the garden just for some personal comfort for yourself and I honestly don’t mind… it’s isolating we are living in a big building; we are not seeing each other.*”

Within families, the shared use of private and public natural environments brought family members closer together. Similarly, for those who were shielding or confined to their home environment, sharing photos of the garden replaced normal face-to-face interactions and supported familial connection:

Hannah: “*I can send photos to my son and saying, ‘look how the garden is’ and they might send pictures back of their garden and say, ‘this is what we have got at the moment”. So, we keep in touch like that… it keeps you connected.*”

Nature also became interwoven into conversations where it replaced other conversational topics typical of pre-COVID times, bringing a positive focus:

Andy: “*one my friends the other day said… ‘I don’t know what we would have done without the gardens. What would we have talked about?’ I think we would probably end up talking about how depressed we were or how anxious we were… In terms of a positive thing to talk about what can be more positive than talking about the garden.*”

In this way, sharing nature through conversation, photos, garden space and produce was integral in supporting social interactions and social connections when physical contact with others was restricted due to health vulnerability.

#### 3.3.2. Subtheme: Nature Connections

A heightened awareness of the natural world, ‘feeling at one’ with nature or experiencing wonder and awe was also experienced by some through connecting directly with nature during lockdown:

Wendy: “*at the start of the lockdown I guess it was the time I felt it most. … it was extraordinary to be a witness to the change in landscape around us. I got out early in the morning to go for a walk before I sat and worked in front of my computer, and all you could see was birds, or the sheep. I actually made a little recording of it because I just thought it was just so amazing.*”

For others, nature facilitated time to be alone and to connect with oneself. Participants often identified specific types of natural environments, e.g., blue spaces and mountains, as “*just me*”. Spending time alone in natural environments was central in nurturing this connection with oneself:

Lori: “*being in a terraced house with the three of us with no actual garden to speak of, was quite restricting so it was kind of a nice space to get out and have some time alone… calm and just feeling a bit more, a bit more like me.*”

As reflected by Wendy and Lori, nature often facilitated a deeper connection with oneself as a result of a more direct connection with nature through nurturing plants, “*communing with the silence*” or “*touching*” the soil:

Karen: “*I don’t know why that makes a difference, but having… your hands in compost, in soil, handling plants, I guess it connects you to nature, more than just looking at it, you are there, you are with it, you are touching it, you are doing something to it.*”

This interconnection between identity and nature was further evident where nature served as an important symbol of cultural identity (e.g., growing shamrock). For Wendy, nature held significance and meaning at a time when their “*[St Patrick’s Day event] was cancelled*” due to COVID-19. Through growing shamrock, Wendy was able to connect to her culture and heritage, something important to her at that time: “*I wanted to keep a bit of that shamrock that I grew in a pandemic when we couldn’t celebrate where we came from*”.

This sense of nostalgia was also evident for others where a greater awareness of nature over COVID-19 reconnected participants with earlier memories of nature. This was expressed by some as a desire to capture and create a record of nature, as a reflection on the past and the fostering of resilience during the pandemic. For Wendy, creating a record was related to a future point in time, as something to reflect on with the next generation:

Wendy: “*I am intending to put together a collection of those country lane walks and the succession of plants as they came up. I think that would be a nice thing to do, as kind of a record of the pandemic… Well, I don’t know, how is the world going to turn out here. Will our grandchildren ask us what we did here? it would be kind of nice to have that record.*”

#### 3.3.3. Subtheme: Noticing Nature

Participants reported noticing nature more during the COVID-19 lockdown. This included nature sounds, scents, wildlife, plants and vegetation, and seasonal changes. Noticing nature led to greater sense of nature connectedness, where time and opportunity were salient factors in nature engagement, in particular private greenspace where participants were spending more time. Participants spoke of the positive impact of noticing nature and how this differed during COVID-19:

Kevin: “*you see the changes, because you are I suppose examining the garden in much more minute detail, it’s that minutia that …begins to impact on you. So, you see things changing but perhaps in the past you wouldn’t have noticed because you weren’t looking at it in quite so much detail.*”

Noticing nature, specifically nature sounds and bird song, was frequently referenced in relation to decreased traffic volume and noise pollution due to lockdown restrictions. Many referred to the stark silence and lack of background noise at the start of the COVID-19 lockdown, which coincided with an increased awareness of nature sounds and resulting feelings of peace and calm:

Sally: “*quite early on in lockdown we didn’t get the traffic noise and I just would sit in the garden, and I would just video it because there was this stunning blue sky and then the green of the garden and you could hear all the birds singing and you couldn’t hear the traffic and the sky was so clear … it was really peaceful.*”

Being more aware of the beauty of nature was often referenced, where seasonal changes brought newness, colour and growth, and evoked feelings of “*pleasure and unexpectedness*” [Katie]. This was most often contrasted with a more restricted and confined experience associated with the pandemic.

#### 3.3.4. Subtheme: Enhanced Appreciation of Nature

Noticing nature led to a greater appreciation of nature. Participants expressed how the pandemic had increased their awareness of the importance of nature, including become more environmentally conscientious. Florence highlights this appreciation when saying: “*it has been a godsend having a garden*” and goes on to explain: “*you appreciate it more because you are looking at it through different eyes…COVID has changed how a lot of people see things*.”

Looking at nature through “*different eyes*” indicates a sense of changed priorities, also echoed by Rachel, where a greater appreciation of nature in supporting her health and wellbeing during the pandemic resulted in a shift in how she perceived herself: “*I think that is something I will take forward with me, is the fact that I am important, I am worth that time… your wellbeing is more important*.”

A greater appreciation and value assigned to nature alongside an increased awareness of the interconnectedness between the human and natural world often led to other significant changes, e.g., visiting natural environments more often, plans to holiday in local natural environments, engaging in more pro-environmental behaviours, buying local produce, and supporting local businesses. While most engaged in pro-environmental behaviours to begin with, for many these behaviours alongside pro-social behaviours increased. For some, the experience of the pandemic acted as a confirmation of a more long-term desire to live closer to nature with several wanting to move out of urban settings to more rural locations: “*we want more of this peace and quiet, we want more of the simple life*” [Andy].

Related to a greater appreciation of natural environments was participants’ heightened awareness of their “*good fortune*”, and luck in being “*wealthy in the space we have*” [Sally]. Access to outdoor space was seen as a fundamental right for everyone, reinforced by participants’ appreciation of the role of natural environments (in particular, private greenspace), and associated benefits to their health and wellbeing. This valuing of nature was also linked to an increased awareness of others and their different experiences. Connected to this was the need to preserve such spaces for the future benefits of others and to give something back in appreciation of their positive experiences:

Sally: “*I also appreciate [natural environments] more for other people in the flats, and public green spaces are the only green spaces they have and their experience of COVID has probably been very different to ours. So, it has made me more determined to preserve our green spaces.*”

### 3.4. Theme 4: Therapeutic Nature

This theme captures the therapeutic benefits of nature (both psychological and physical) experienced by participants. Integral to these therapeutic benefits were the sensory stimulation that connecting with nature provided. Mindfulness was also central in supporting mental health and wellbeing through facilitating an immersion in nature. Participants referred to private greenspaces specifically, as being a “*life-saver*” and a “*life-line*” and not being able to cope without it.

#### 3.4.1. Mental Health

Nature supported participants through various psychological pathways. Natural environments, including private greenspace and public green and blue spaces, provided opportunities for relaxation; functioning as calm environments in contrast to the realities associated with COVID-19:

Sally: “*it was really peaceful… any time that I was feeling a bit tense about everything I would just go into the garden for a little bit, and I could just feel it all dissolve away.*”

As reflected by Sally, engaging with natural environments was central in managing stress and a vital coping mechanism during COVID-19. However, for others, daily access to outdoor space beyond the confines of the home environment was important for their mental health and a fundamental green prescription:

William: “*If I didn’t have a space outside to get into, if I didn’t have the garden and if I didn’t have the …nature reserve just down the road, I would have crashed badly and I know for a fact … I would have been back on anti-depressants, and I came off them two years ago and I haven’t been back since.*”

Several participants acknowledged the negative consequences for their mental health if they were not able to access the outdoors, specifically describing how they would not have been able to cope without it:

Sally: “*I would have found it very difficult to cope if I hadn’t had that release of being able to get out of the house, getting to somewhere that was just pretty in its own right that made you think* *‘oh that’s lovely’ or* *‘doesn’t that bird sound really good’. I think the impact on sort of maintaining my sanity over that time, it was just massive really.*”

For those who were shielding or considered high risk, natural environments had an added value in supporting mental health and wellbeing. Even spending a limited time in private greenspace was significant, where nature provided a sense of buoyancy during a time of uncertainty and imposed restrictions. This was reflected by Philip where gardening became central in supporting his wellbeing: “*it is quite uplifting*”, despite not liking gardening prior to the pandemic.

#### 3.4.2. Subtheme: Physical Health

For some participants, engaging with natural environments was important in managing their physical health conditions and supporting better sleep. Being active in the garden, through gardening or physical exercise, supported participants cope with chronic pain:

Barbara: “*I am taking pain killers every day to sort of manage [chronic back pain]. The gym is like a really important aspect of my day-to-day wellbeing to keep it under control. So, when gyms stopped, I thought ‘no I can really feel the fact of not going to the gym, it’s not helping me here’. So, having, having the space to go out in the garden and do something has been really helpful.*”

Stacey also made the decision to get out despite it being considered high risk: “*very difficult decision to make because we were all scared*”, where being in public natural spaces were central in managing her stress, and chronic pain. Similarly for Jon, natural environments played a pivotal role in his stroke rehabilitation. Being in nature supported his mental health immediately after his stroke, while providing him with resources and strategies such as “*strolling around the garden and observing wildlife, and going very slowly*”, which “*was a big help in my rehabilitation*”.

#### 3.4.3. Subtheme: Multi-Sensory Engagement

A sensory experience of nature was central to the therapeutic benefits for some. Those who had been shielding or were house-bound due to their health vulnerability reported a heightened awareness and sensory experience of nature. Aromatic smells, nature sounds, and the beauty of nature were key to enhancing mood:

Florence: “*going out for a walk on the nature reserve, literally you smell so much more, you see so much more, and you hear so much more. Because you have been cooped up for so long, it’s like having hyper-sensitive hearing and hyper-sensitive sense of smell, it’s like the first time you have ever done anything like that. And you just take it all in.*”

While the colour and smells of plants (indoor and outdoor) were important, the diversity of colour in domestic gardens was particularly powerful, stimulating positive emotions, such as joy. Wendy also references a therapeutic impact at a cognitive level, where contrasts were drawn between natural environments and technology:

Wendy: “*Even sitting here now I am looking out the door into a very green world and it just seems to be massage for the brain cells something like that. Especially when you’ve… away from the computer and hard lines and lights.*”

As reflected by Wendy and others, this awareness of the sensory and psychological impacts of nature during lockdown, generated a deeper appreciation and understanding of the therapeutic benefits of nature in participants’ lives.

#### 3.4.4. Subtheme: Mindfulness

The therapeutic benefits of nature were also referenced in relation to mindfulness, where engaging with nature directly or creatively allowed participants to immerse themselves in a different world beyond that of their immediate personal reality:

Florence: “*You can lose yourself more when you are in such an open space and your mind just empties.*”

Similarly, engaging with nature through creativity had a therapeutic benefit through facilitating a sense of reprieve from the realities of COVID-19. Mindfulness was central in realising this benefit where nature provided an alternative focus and captured participants’ attention to detail:

Andy: “*You are totally focused in that moment of that thing, so you are looking at the colours you’re using, you’re looking at the shapes. It’s that intense mindfulness. It’s that intensity of observation. It’s the intensity of thinking well what colours exactly are on the outside of that petal on that flower. How is that flower constructed and while you’re doing that while you’re minutely identifying that you’re not thinking of anything else.*”

In a similar way, connecting with nature facilitated a mental respite from COVID-19 related thoughts and worries, through orientating participants to the present and providing a positive focus thorough immersion in the natural world:

Jon: “*to get out in the garden and just completely immerse myself in bug spotting and looking at botany and bird watching, I would just completely forget about all of that and be in my own little world, which I think was great because it just gave my brain a pause.*”

## 4. Discussion

The present research is the first qualitative exploration of how those with a pre-existing health condition perceived and interacted with nature in relation to their health and wellbeing during the first UK COVID-19 lockdown. The findings show the importance of nature in supporting health and wellbeing through engagement with private and public natural environments, providing opportunities for restoration, nature connection as an important pathway, and the therapeutic benefits of nature. Findings are discussed in relation to relevant themes in the literature, including research on health-supporting environments, micro-restorative nature opportunities, and possible implications for policy.

### 4.1. Health Supporting Environments

The superordinate themes ‘COVID-19 versus nature’ and ‘nature as an extension and replacement’ revealed that private and public natural environments were thought to support positive health in a number of ways.

#### 4.1.1. Private Outdoor Spaces

The present research showed that access to private outdoor space was perceived as beneficial to health and wellbeing for those with a pre-existing health condition during the pandemic and provides further support for private green spaces as health-supporting environments [55,56,69,70]. It also extends the literature by exploring the types of nature engagement and ways in which they support health and wellbeing in a vulnerable subpopulation during a public health crisis. Specifically, private garden use during the pandemic was considered essential for psychological health and wellbeing (i.e., supporting positive mental health, reducing stress, increasing positive mood, promoting work-life balance), physical health (i.e., managing pain, improving sleep, supporting weight loss and fitness) and social wellbeing (i.e., sustaining social connections and supporting social interactions) through active (i.e., gardening, physical exercise), passive (i.e., noticing nature, watching wildlife, listening to nature sounds) and quasi-passive nature engagement (i.e., touching soil and plants, smelling flowers, taking photos and videos of the garden).

These findings are in line with research showing that gardens were more frequently used than other types of natural environments during the first lockdown [71], with greater frequency of use related to positive health and wellbeing outcomes [55,70,71]. In addition, there is robust support for positive health outcomes associated with gardening [23]. The findings also provide support for private gardens as a valuable physical activity support and add to previous research in this area by exploring the various types of private outdoor space used to support physical activity during lockdown [56,70]. Moreover, the findings of the present research highlight the psychological and physical health benefits associated with engaging in physical activity in private greenspace for those with pre-existing health conditions. While participants were not consciously choosing gardening as ‘exercise’, many were aware of the resulting health benefits, which may have reinforced their motivation to continue with gardening.

In the present study, many participants were anxious about the risk of accessing public natural spaces due to their vulnerability to COVID-19; findings reflected elsewhere among cancer patients [72]. Previous research has identified private outdoor spaces as an important resource in the nature–health relationship, which may not be replaced by other urban natural environments [70]. The security and privacy of private gardens [73] and the opportunity to create and express one’s own identity through private garden space [74] are thought significant in realising such benefits. While some chose to engage with public natural spaces for additional benefits (i.e., psychological stimulation, greater sense of escape, personal reflection), there was evidence of a substitution effect for private greenspace (instead of public natural environments) [75] in those who were vulnerable through a health diagnosis. The findings add to previously inconsistent findings on the substitution effect of private greenspace [76], through identifying health vulnerability as a salient factor in participants’ relationship and use of greenspaces.

#### 4.1.2. Active and Passive Nature Engagement

The present research showed that both active and passive nature engagement were important to participants. Those who engaged in gardening benefited from a sense of industriousness, focus, challenge, achievement, and reward. Others with significant health challenges found ways to adapt to working in their garden (e.g., use of tools, short duration, aware of body position when gardening), where gardening activities were highly valued. Most participants engaged in passive nature engagement (e.g., sitting, being in nature, observing and appreciating nature views, listening to nature sounds) in private and shared gardens, and public natural spaces. While sitting in the garden was not always as consciously valued as active nature engagement, there was evidence of a shift towards greater recognition of the value of ‘just sitting’ and being in the garden, which was not necessarily recognised pre-COVID. Previous research on passive nature engagement in gardens provides support for health and wellbeing outcomes for vulnerable populations for both outdoor [77,78] and indoor settings [79,80]. Similar to previous research [59,81,82,83,84], participants in the present research considered private garden space to be restorative during COVID-19, through direct and immediate access to sunshine and fresh air, inducing feelings of calm/peace, reducing stress, improving mood and cognition.

#### 4.1.3. Local Natural Environments

The findings of this research demonstrate that for some people with existing health conditions, public natural environments were essential for psychological stimulation and mental space during COVID-19, in addition to supporting opportunities for exercise and social interaction. Public natural environments offered a greater sense of escape and private thinking time away from the home environment and from media relating to COVID-19. Local natural environments which were considered safe were not only essential in supporting mental health and wellbeing for those with a pre-existing health condition but were significant through changing perceptions of the value of such spaces. This aligns with research showing that easily accessible urban nature facilitated greater positive health benefits [85] and was more valued and appreciated [86]. Local natural environments were also considered more biodiverse by participants, providing psychological stimulation, and facilitating a greater sense of ‘being away’ from the home environment. Research has also highlighted the role of diverse natural environments in supporting life satisfaction [60], where more biodiverse environments provide a richer flora and fauna, thus enhancing psychological wellbeing [87].

Smaller green spaces may also be a more suitable and accessible UGS for vulnerable subpopulations [88], where public greenspaces (i.e., parks) were often considered unsafe during the pandemic by participants who had a health vulnerability. Similarly, for one participant, an allotment was a valuable greenspace which afforded a sense of psychological and physical safety. The WHO identified that “*even small-scale greening interventions can deliver health, social and environmental benefits in a cost-efficient way, where not many public health interventions can achieve all of this*” [2]. Allotments have potential as small-scale green interventions, offering a health promoting activity for the general population and supporting health and wellbeing for clinical populations [89].

#### 4.1.4. Therapeutic Landscapes

Therapeutic landscapes where “*physical and built environments, social conditions and human perceptions combine to produce an atmosphere which is conducive to healing*” [90] (p. 96), may be especially relevant for those with a health condition [91]. Affective experiences of space and attachment to place may be important, in addition to the landscape’s physical qualities [91,92]. Everyday landscapes (e.g., home, community) may have become significant therapeutic landscapes during COVID-19 for those with a pre-existing health condition, where the home environment acted as a safe space in supporting health [93], and local greenspaces cultivated health and wellbeing [78]. Emotional geographies, such as place attachment, may be central in how private and local natural spaces act as a therapeutic landscape for those with a pre-existing health condition. The underlying processes that sustain place attachment (i.e., place definition, place dependence, place bonding, place interaction, and place identity) [94] alongside a heightened sensory, affective, and experiential experience of nature may have resulted in an interweaving of person and place to create a more embodied experience of nature as described by the participants in the present study.

The study findings illustrate the importance of private outdoor spaces in helping participants who were shielding or house-bound due to their health vulnerability cope with the stress of COVID-19; private outdoor space was considered a lifeline. This agrees with research that found nature supported positive affect, and the buffering effect of accessible greenspace and nature views were especially important for those experiencing more restricted lockdowns [95]. It also aligns with the literature on nature’s role in building psychological resilience and buffering stress in times of crisis [96,97].

### 4.2. Micro-Restorative Opportunities

Opportunities for brief nature exposure may act as a viable micro-restorative experience as reflected by the participants in this research, which accords with evidence that just 5–20 min of nature exposure can increase positive affect, decrease negative affect, and reduce stress [98,99]. Such opportunities may be more important during times of crisis, suggesting a need for more regular micro-restoration to manage ongoing stress associated with a pandemic [100], particularly in the most vulnerable. This may have important implications for public health where research has found that people underestimate the degree to which even brief contact with natural environments can improve mood [101].

#### 4.2.1. Digital Nature

The findings of the present research confirm a significant increase in digital nature engagement during lockdown, through online nature activities [53], nature websites [39], and webcam travel (i.e., visiting place-based webcams online) [50]. Digital nature engagement provided a valuable opportunity for those with pre-existing health conditions to access other natural spaces, enabling a sense of escape and creating feelings of nostalgia through facilitating connections to familiar or preferred places [50].

Nostalgia has been shown to be important in the development of psychological resilience, through the creation of memories that connect us to places which hold meaning and are integral to our identity [102]. In this way, digital nature engagement (i.e., nature videos, photos, webcams) can foster psychological resilience during lockdowns, particularly for those who may experience greater restrictions due to health vulnerability.

Similar to engagement with ‘real’ nature, the research findings indicate benefits of digital nature engagement to facilitate restoration (and associated psychological benefits), and for social connection, where the use of blogs or virtual meetings to support the indirect social benefits of gardening has been proposed [56]. The study findings concur to some extent; that digital nature may augment or be a viable alternative when in-vivo nature is unavailable [103,104]. However, this may not be an adequate replacement for ’real’ nature experiences, where the inability to access certain natural environments prompted ambiguous feelings or because digital nature fails to provide a complete sensory experience.

#### 4.2.2. Nature Views

Windows have psychological importance, providing sensory stimulation, meaningful contact, important information which connects the immediacy of our indoor environment to the broader world beyond us [105]. The study findings showed that nature views were considered important in providing a sense of normality and supporting wellbeing during a period of significant and unprecedented change and altered working conditions. Nature views can provide micro-restorative experiences and associated improvements in affect, where the buffering effect of accessible nature and nature views were especially pertinent for those experiencing stricter lockdowns [95]. Similar to previous research in hospitalised patients, the findings showed that a view of nature provided a sense of normality; a reminder of the world to which participants belong, which could foster hope of the world they can eventually return to [105]. In the context of the pandemic, nature also offered a sense of hope and normality for those experiencing greater restrictions due to their health vulnerability. For example, observing seasonal colours, changes, and new growth contrasted with the uncertainty and fear of COVID-19. In this way, nature views may be an important nature-based intervention for mental health [46]. They can induce a feeling of ‘being away’, provide respite even briefly, and support sense-making and meaning. While most participants in the present study had immediate access to a private outdoor or shared green space, nature views to support restoration may be especially relevant for those who are house-bound [106], have limited physical capacities [37,105], or work from home [107,108].

#### 4.2.3. Nature Sounds

During the first COVID-19 lockdown, participants reported noticing more nature sounds and hearing louder bird song, as reported elsewhere [109]. Bird song may provide valuable opportunities for restoration [110], where certain bird sounds are associated with recovery from mental fatigue and stress [111]. Specifically, bird sounds that generate positively valenced appraisals (e.g., pleasant, melodic) can be restorative, while those that generate negatively valenced appraisals (e.g., unpleasant, stressful) are considered unhelpful [112].

Previous research has also shown that, compared to urban soundscapes (e.g., traffic, café ambiance, machinery), natural soundscapes (e.g., birdsong, water, insects, and wind) were preferred, but did not significantly improve mood [113]. However, natural sounds did significantly improve cognitive performance, highlighting that even brief exposure to natural sounds can have a restorative impact by improving directed attention functioning. During COVID-19, web-based relaxation practices of guided breathing, guided body scan and natural sounds resulted in a positive improvement in perceived relaxation, psychomotor activation/stress, and preoccupation related to COVID-19, with superior benefits for guided breathing and body scan in reducing stress [114]. Despite some inconsistency, there is convergent evidence of the benefits of nature sounds in supporting restoration and, therefore, a greater awareness and appreciation of nature sounds among the present research sample during lockdown provides support for these benefits.

### 4.3. Policy Implications

#### 4.3.1. Health-Supporting Environments

This research highlights private outdoors space as an important health supporting environment for vulnerable subpopulations. While previous research identified that further health promotion activities are required to specifically target those who would most benefit from private greenspace [70], the findings of the present research (i.e., ‘Therapeutic Nature’ and ‘Nature Connectedness’ superordinate themes) suggest ways in which private natural environments can support health and wellbeing in those with a pre-existing health condition (e.g., opportunities to engage in physical activity; sensory and mindful experiences to support mental health and wellbeing; social engagement and interactions). This may have implications for both policy and public health messaging where the use of private outdoor space can be advocated as an important measure in mitigating the negative effects of any future lockdowns.

Challenges in engaging with public natural environments for those with a pre-existing health condition are highlighted by the findings of this research. Understanding how UGS should be optimally designed, used, and promoted to best support population health, is vital [115]. The inclusion of vulnerable subpopulations, disadvantaged communities, and those most in need of restoration in the decision-making process of UGS is key to ensure equitable health outcomes for all population groups in light of significant demographic, societal and environmental changes [33].

The role of ‘nature connectedness’ in the nature–health relationship is also highlighted by this research, where greater nature connectedness (more than time spent in nature) was central to realising both hedonic and eudaimonic well-being benefits [116]. Therefore, activities that promote greater nature connectedness could be integrated into nature programmes, UGS design, and incorporated into public health messaging for private and public natural environment engagement. Specifically, these could focus on the proposed five ways to promote nature connection, as reflected by the findings of this research: emotion (e.g., taking a moment to feel calm in nature), senses (e.g., listening to birdsong), beauty (e.g., taking a photo of a flower), meaning (e.g., creating a nature project that is linked to cultural identity) and compassion (e.g., growing a wildflower meadow to support wildlife) [117].

#### 4.3.2. Green Social Prescribing and Green Care

The present research also shows that artistic and digital nature engagement may serve as viable health supporting interventions through facilitating mindfulness, social engagement, and nostalgia. Artistic nature may have relevance for green social prescribing involving group interventions indoors, outdoors, or online. Digital technologies may offer new opportunities to combine nature and social engagement through social media platforms, online groups, and blog writing. While there is a good evidence-base for the efficacy of nature-based interventions in addressing mental health and social care issues [32,118], further research is required to evaluate green prescription interventions (i.e., nature-based interventions for those with a defined psychological or physical health need) to determine the mechanisms and contexts in which these interventions are most effective [119].

### 4.4. Future Research

All participants in the present research had access to garden space (private and/or shared) and the first UK lockdown occurred during a period of sustained good weather. This may have reduced the need for indoor greening, which might be relevant for those who do not have immediate access to natural environments or who spend most of their time indoors [44]. Specifically, exploring the therapeutic impacts of passive and quasi-passive indoor nature engagement for vulnerable groups who do not have immediate access to a private greenspace would be valuable in identifying benefits to health and wellbeing (e.g., nursing home residents) [45]. Related to this, research that explores how those in the community living with a pre-existing health condition, but with no access to private outdoor space, engaged with nature during the COVID-19 pandemic would add to the findings of the present research.

### 4.5. Limitations

The limitations of this study are recognised. First, online recruitment limited the qualitative sample to those with internet access and a certain level of technology proficiency. Second, the survey through which the sample was reached showed a bias towards responses from white participants and an over-representation of highly educated people and those from higher socio-economic groups. Therefore, the sample is not representative of the general UK population, although this was not an objective for this qualitative study of individual’s experiences. Third, the intersectionality of ethnicity, socio-economic status, and those with no access to private greenspaces, in relation to health inequalities has not been explored, which may have additional implications for understanding health enabling and supporting environments.

## 5. Conclusions

This was the first qualitative exploration of how people with pre-existing health conditions perceived and engaged with nature during the first UK COVID-19 lockdown. The present research extends the evidence-base beyond patterns of nature engagement to a deeper understanding of how those with existing health conditions perceived and interacted with nature in relation to their health and wellbeing during the first UK lockdown. The findings show the importance of nature in supporting health and wellbeing through engagement with private and public natural environments, providing opportunities for restoration, nature connection as important pathway, and the therapeutic benefits of nature. This extends the literature by further highlighting the importance of private outdoor space provision, alongside UGS in urban policy and planning, to protect and promote natural environments as a public health resource for people with poorer health during COVID-19. Micro-restorative nature engagement opportunities are a potentially important means to buffer stress during a crisis and require further exploration and emphasis in public health messaging. The role of artistic and digital nature should be further explored as part of green social prescribing initiatives with potential for groups to connect remotely, and where mindfulness, social engagement and the generation of nostalgia may be key ways to support mental health and wellbeing. Limitations of the present study included a lack of generalisability (although this was not the aim of this qualitative study). Future research could explore intersectionality and health inequalities, indoor greening, and the therapeutic impacts of indoor nature exposure.

## Figures and Tables

**Table 1:** Overview of the 17 participants quoted in the text, including pseudonyms, gender, age, health condition, outdoor space, ethnicity, and employment status.

Name	Gender	Age	Health Condition	Outdoor Space	Ethnicity	Employment Status
Andy	M	58	Asthma	Private medium garden	White	Retired
Kevin	M	61	Cardiovascular	Private medium garden	White	Retired
Barbara	F	45	Chronic pain, Asthma	Private garden (front and back)	White	Working from home
Karen	F	51	Diabetes	Private medium garden	White	Working from home
Sally	F	55	Lupus, nerve pain	Private large garden	White	Working from home
Wendy	F	57	Weakened Immune	Private large garden	White Irish	Working from home
Katie	F	59	Cancer, asthma ^1^	Smallholding	White	Working from home; student
Rachel	F	65	Hypertension, osteoarthritis, asthma	Private garden (canal facing and back garden)	White	Working from home
Florence	F	46	Weakened immune ^1^	Private garden (end of terrace)	White	Student
Stacey	F	75	Arthritis, fibromyalgia	Communal garden	White	Retired
William	M	37	Asthma	Private gravel garden	White	Working from home
Philip	M	69	Weakened immune ^1^	Private large garden	White	Retired
Hannah	F	72	Hypertension	Private garden (front and back)	White	Retired
Lori	F	34	Anxiety	Private yard and private allotment	White	Working from home
Olive	F	66	Breast cancer and stroke	Private small garden and shared communal garden	White	Retired
Susan	F	28	Anxiety	Private garden (parent’s garden)	White	Student
Jon	M	60	Major stroke	Private large garden (meadow, brook and woodlands)	White	Working from home

^1^ Shielding.

**Table 2:** Superordinate themes and subthemes.

Superordinate Theme	Sub-Theme	Sub-Theme	Sub-Theme	Sub-Theme
COVID-19 versus nature	Nature as an escape from COVID-19	Adaptations and considerations	Safety and Control	
Nature as an extension and replacement	The garden as a passive and active space	Public natural spaces as an extension	Micro-restorative nature	
Nature connectedness	Sharing nature	Nature connections	Noticing nature	Enhanced appreciation of nature
Therapeutic nature	Mental health	Physical health	Multisensory engagement	Mindfulness

## Data Availability

The data from this qualitative study are not suitable for sharing beyond that presented in this article. Data are in the form of interview transcripts and contain personal and sensitive data, which present ethical challenges regarding data sharing for secondary analysis e.g., threats to participant anonymity, privacy, and confidentiality; unknown future use of data in relation to original participant consent and ethical approvals [120]. For more information, please contact the corresponding author.

## Supplementary Materials

The following are available online at https://www.mdpi.com/article/10.3390/ijerph19073908/s1, Table S1: Interview Topic Guide; Table S2: Consolidated criteria for reporting qualitative studies (COREQ): 32-item checklist; Figure S1: Illustration of results.

## References

[1] DEFRA (2018). A Green Future: Our 25 Year Plan to Improve the Environment.

[2] WHO (2016). Urban Green Spaces and Health.

[3] Norwood Francis M., Lakhani A., Fullagar S., Maujean A., Downes M., Byrne J., Stewart A., Barber B., Kendall E. (2019). Landscape and Urban Planning A narrative and systematic review of the behavioural, cognitive and emotional effects of passive nature exposure on young people: Evidence for prescribing change. Landsc. Urban Plan..

[4] Mygind L., Kjeldsted E., Hartmeyer R.D., Mygind E., Bølling M. (2019). Immersive Nature-Experiences as Health Promotion Interventions for Healthy, Vulnerable, and Sick Populations? A Systematic Review and Appraisal of Controlled Studies. Front. Psychol..

[5] Tillmann S., Tobin D., Avison W., Gilliland J. (2018). Mental health benefits of interactions with nature in children and teenagers: A systematic review. J. Epidemiol. Community Health.

[6] Cox D.T.C., Shanahan D.F., Hudson H.L., Fuller R.A., Anderson K., Hancock S., Gaston K.J. (2017). Doses of nearby nature simultaneously associated with multiple health benefits. Int. J. Environ. Res. Public Health.

[7] Bowler D.E., Buyung-ali L.M., Knight T.M., Pullin A.S. (2010). A systematic review of evidence for the added benefits to health of exposure to natural environments. BMC Public Health.

[8] Yao W., Zhang X., Gong Q. (2021). The effect of exposure to the natural environment on stress reduction: A meta-analysis. Urban For. Urban Green..

[9] Mygind L., Kjeldsted E., Hartmeyer R., Mygind E., Stevenson M.P., Quintana D.S., Bentsen P. (2021). Effects of Public Green Space on Acute Psychophysiological Stress Response: A Systematic Review and Meta-Analysis of the Experimental and Quasi-Experimental Evidence. Environ. Behav..

[10] Staats H., Kieviet A., Hartig T. (2003). Where to recover from attentional fatigue: An expectancy-value analysis of environmental preference. J. Environ. Psychol..

[11] Stevenson M.P., Schilhab T., Bentsen P. (2018). Attention Restoration Theory II: A systematic review to clarify attention processes affected by exposure to natural environments. J. Toxicol. Environ. Health.

[12] Mcmahan E.A., Estes D. (2015). The Effect of Contact With Natural Environments on Positive and Negative Affect: A Meta-Analysis. J. Posit. Psychol..

[13] Capaldi C.A., Dopko R.L., Zelenski J.M. (2014). The relationship between nature connectedness and happiness: A meta-analysis. Front. Psychol..

[14] Bratman G.N., Daily G.C., Levy B.J., Gross J.J. (2015). Landscape and Urban Planning: The benefits of nature experience: Improved affect and cognition. Landsc. Urban Plan..

[15] Daniels S., Clemente D.B.P., Desart S., Saenen N., Sleurs H., Nawrot T.S., Malina R., Plusquin M. (2022). Introducing nature at the work floor: A nature-based intervention to reduce stress and improve cognitive performance. Int. J. Hyg. Environ. Health.

[16] Corazon S.S., Sidenius U., Poulsen D.V., Christo M. (2019). Psycho-Physiological Stress Recovery in Outdoor Nature-Based Interventions: A Systematic Review of the Past Eight Years of Research. Int. J. Environ. Res. Public Health.

[17] Jo H., Song C. (2019). Physiological Benefits of Viewing Nature: A Systematic Review of Indoor Experiments. Int. J. Environ. Res. Public Health.

[18] Wray A., Martin G., Ostermeier E., Medeiros A., Little M., Reilly K., Gilliland J. (2020). Evidence synthesis Physical activity and social connectedness interventions in outdoor spaces among children and youth: A rapid review. Health Promot. Chronic Dis. Prev. Can. Res. Policy Pract..

[19] Alaimo K., Beavers A.W., Crawford C., Snyder E.H., Litt J.S. (2016). Amplifying Health Through Community Gardens: A Framework for Advancing Multicomponent, Behaviorally Based Neighborhood Interventions. Curr. Environ. Health Rep..

[20] Ward Thompson C., Aspinall P., Roe J., Robertson L., Miller D. (2016). Mitigating stress and supporting health in deprived urban communities: The importance of green space and the social environment. Int. J. Environ. Res. Public Health.

[21] Kaplan R. (2001). The nature of the view from home psychological benefits. Environ. Behav..

[22] Dennis M., James P. (2017). Evaluating the relative influence on population health of domestic gardens and green space along a rural-urban gradient. Landsc. Urban Plan..

[23] Soga M., Gaston K.J., Yamaura Y. (2017). Gardening is beneficial for health: A meta-analysis. Prev. Med. Rep..

[24] McMichael A.J. (2000). The urban environment and health in a world of increasing globalization: Issues for developing countries. Bull. World Health Organ..

[25] Colley K., Currie M., Hopkins J., Melo P. (2016). Access to Outdoor Recreation by Older People in Scotland: Report for Rural Communities Research, Rural and Environment Science and Analytical Services (RESAS) Division, The Scottish Government.

[26] Donaldson G.C., Wilkinson T.M.A., Hurst J.R., Perera W.R., Wedzicha J.A. (2005). Exacerbations and time spent outdoors in chronic obstructive pulmonary disease. Am. J. Respir. Crit. Care Med..

[27] Segura-Jiménez V., Álvarez-Gallardo I.C., Estévez-López F., Soriano-Maldonado A., Delgado-Fernández M., Ortega F.B., Aparicio V.A., Carbonell-Baeza A., Mota J., Silva P. (2015). Differences in sedentary time and physical activity between female patients with fibromyalgia and healthy controls: The al-Ándalus project. Arthritis Rheumatol..

[28] Galea K.S., Hurley J.F., Cowie H., Shafrir A.L., Sánchez Jiménez A., Semple S., Ayres J.G., Coggins M. (2013). Using PM2.5 concentrations to estimate the health burden from solid fuel combustion, with application to Irish and Scottish homes. Environ. Health.

[29] Mcsweeney J., Rainham D., Johnson S.A., Sherry S.B., Singleton J. (2015). Indoor nature exposure (INE): A health-promotion framework. Health Promot. Int..

[30] Yin J., Zhu S., MacNaughton P., Allen J.G., Spengler J.D. (2018). Physiological and cognitive performance of exposure to biophilic indoor environment. Build. Environ..

[31] Phelps C., Butler C., Cousins A., Hughes C. (2015). Sowing the seeds or failing to blossom? A feasibility study of a simple ecotherapy-based intervention in women affected by breast cancer. Ecancermedicalscience.

[32] Bragg R., Atkins G. (2016). A Review of Nature-Based Interventions for Mental Health Care (NECR204). Natural England Commissioned Reports, Number 204. http://publications.naturalengland.org.uk/publication/4513819616346112.

[33] Darcy P.M., Jones M., Gidlow C., Donnelly A.A., MacIntyre T.E. (2019). Affective responses to natural environments: From everyday engagement to therapeutic impact. Physical Activity in Natural Settings.

[34] Beukeboom C.J., Langeveld D., Tanja-Dijkstra K. (2012). Stress-reducing effects of real and artificial nature in a hospital waiting room. J. Altern. Complem. Med..

[35] Diette G.B., Lechtzin N., Haponik E., Devrotes A., Rubin H.R. (2003). Distraction therapy with nature sights and sounds reduces pain during flexible bronchoscopy: A complementary approach to routine analgesia. Chest.

[36] Tanja-Dijkstra K., Pahl S., White M.P., Auvray M., Stone R.J., Andrade J., May J., Mills I., Moles D.R. (2018). The Soothing Sea: A Virtual Coastal Walk Can Reduce Experienced and Recollected Pain. Environ. Behav..

[37] Ulrich R. (1984). View through a window may influence recovery. Science.

[38] (2020). Google COVID-19 Community Mobility Reports. https://www.google.com/covid19/mobility/.

[39] ONS (2021). How Has Lockdown Changed Our Relationship with Nature?. https://www.ons.gov.uk/economy/environmentalaccounts/articles/howhaslockdownchangedourrelationshipwithnature/2021-04-26.

[40] Greenspace Scotland Parks and Greenspace in Scotland—COVID-19 Survey. https://www.greenspacescotland.org.uk/news/parks-and-greenspaces-in-scotland-covid-19-survey.

[41] Douglas M., Katikireddi S.V., Taulbut M., McKee M., McCartney G. (2020). Mitigating the wider health effects of covid-19 pandemic response. BMJ.

[42] Geary R.S., Wheeler B., Lovell R., Jepson R., Hunter R., Rodgers S. (2021). A call to action: Improving urban green spaces to reduce health inequalities exacerbated by COVID-19. Prev. Med..

[43] ONS (2020). One in Eight British Households Has No Garden. https://www.ons.gov.uk/economy/environmentalaccounts/articles/oneineightbritishhouseholdshasnogarden/2020-05-14?hootPostID=b2b740f2d1d59bcf7120bf226074fbcd.

[44] Pérez-Urrestarazu L., Kaltsidi M.P., Nektarios P.A., Markakis G., Loges V., Perini K., Fernández-Cañero R. (2021). Particularities of having plants at home during the confinement due to the COVID-19 pandemic. Urban For. Urban Green..

[45] Spano G., D’Este M., Giannico V., Elia M., Cassibba R., Lafortezza R., Sanesi G. (2021). Association between indoor-outdoor green features and psychological health during the COVID-19 lockdown in Italy: A cross-sectional nationwide study. Urban For. Urban Green..

[46] Soga M., Evans M.J., Tsuchiya K., Fukano Y., Soga M., Evans M.J., Tsuchiya K., Fukano Y. (2021). A room with a green view: The importance of nearby nature for mental health during the COVID-19 pandemic. Ecol. Appl..

[47] Hartig T., Mitchell R., De Vries S., Frumkin H. (2014). Nature and health. Annu. Rev. Public Health.

[48] Kaplan S. (1995). The Restorative Benefits of Nature: Toward an Integrative Framework. J. Environ. Psychol..

[49] De R., Pandey N., Pal A. (2020). Impact of digital surge during COVID-19 pandemic: A viewpoint on research and practice. Int. J. Inf. Manag..

[50] Jarratt D. (2021). An exploration of webcam-travel: Connecting to place and nature through webcams during the COVID-19 lockdown of 2020. Tour. Hosp. Res..

[51] Xu S., Murrell G., Golding S.E., Gatersleben B., Scarles C., White E.V., Willis C., Wyles K.J., Freeman E.L. (2021). # Springwatch # WildMorningswithChris: Engaging With Nature via Social Media and Wellbeing During the COVID-19 Lockdown. Front. Psychol..

[52] The National Trust (2020). Discover Small Moments of Joy in Nature. The National Trust. https://www.nationaltrust.org.uk/features/discover-small-moments-of-joy-in-nature.

[53] (2020). The Wildlife Trusts over 1000 Care Homes Sign-Up to Go Wild This June. The Wildlife Trusts. https://www.wildlifetrusts.org/news/over-1000-care-homes-sign-go-wild-june.

[54] Richardson M., Mcewan K. (2018). 30 Days Wild and the Relationships Between Engagement With Nature’s Beauty, Nature Connectedness and wellbeing. Front. Psychol..

[55] Corley J., Okely J.A., Taylor A.M., Page D., Welstead M., Skarabela B., Redmond P., Cox S.R., Russ T.C. (2021). Home garden use during COVID-19: Associations with physical and mental wellbeing in older adults. J. Environ. Psychol..

[56] Theodorou A., Panno A., Carrus G., Carbone G.A., Massullo C., Imperatori C. (2021). Stay home, stay safe, stay green: The role of gardening activities on mental health during the COVID-19 home confinement. Urban For. Urban Green..

[57] Burnett H., Olsen J.R., Nicholls N., Mitchell R. (2021). Change in time spent visiting and experiences of green space following restrictions on movement during the COVID-19 pandemic: A nationally representative cross-sectional study of UK adults. BMJ Open.

[58] Ugolini F., Massetti L., Calaza-Martínez P., Cariñanos P., Dobbs C., Ostoic S.K., Marin A.M., Pearlmutter D., Saaroni H., Šaulienė I. (2020). Effects of the COVID-19 pandemic on the use and perceptions of urban green space: An international exploratory study. Urban For. Urban Green..

[59] Oswald T.K., Rumbold A.R., Kedzior S.G.E., Kohler M., Moore V.M. (2021). Mental health of young australians during the COVID-19 pandemic: Exploring the roles of employment precarity, screen time, and contact with nature. Int. J. Environ. Res. Public Health.

[60] Chang C.C., Oh R.R.Y., Le Nghiem T.P., Zhang Y., Tan C.L.Y., Lin B.B., Gaston K.J., Fuller R.A., Carrasco L.R. (2020). Life satisfaction linked to the diversity of nature experiences and nature views from the window. Landsc. Urban Plan..

[61] Larkin M., Shaw R., Flowers P., Larkin M., Shaw R. (2019). Multiperspectival designs and processes in interpretative phenomenological analysis research. Qual. Res. Psychol..

[62] Smith J.A., Osborn M. (2007). Pain as an assault on the self: An interpretative phenomenological analysis of the psychological impact of chronic benign low back pain. Psychol. Health.

[63] Smith J.A., Osborn M. (2015). Interpretative phenomenological analysis as a useful methodology for research on the lived experience of pain. Br. J. Pain.

[64] Pietkiewicz I., Smith J.A., Pietkiewicz I., Smith J.A. (2014). A practical guide to using Interpretative Phenomenological Analysis in qualitative research psychology. Psychol. J..

[65] Braun V., Clarke V. (2021). Can I use TA? Should I use TA? Should I not use TA? Comparing reflexive thematic analysis and other pattern-based qualitative analytic approaches. Couns. Psychother. Res..

[66] Smith J.A., Flowers P., Larkin M. (2009). Interpretative Phenomenological Analysis: Theory, Method and Research.

[67] Callary B., Rathwell S., Young B.W. (2015). Insights on the Process of Using Interpretive Phenomenological Analysis in a Sport Coaching Research Project. Qual. Rep..

[68] Dodgson J. (2019). Reflexivity in Qualitative Research. J. Hum. Lact..

[69] Brindley P., Jorgensen A., Maheswaran R. (2018). Domestic gardens and self—Reported health: A national population study. Int. J. Health Geogr..

[70] De Bell S., White M., Griffiths A., Darlow A., Taylor T., Wheeler B., Lovell R. (2020). Spending time in the garden is positively associated with health and wellbeing: Results from a national survey in England. Landsc. Urban Plan..

[71] White E.V., Gatersleben B.C., Wyles K., Murrell G., Golding S.E., Scarles C.E., Xu S. (2021). Gardens & Wellbeing during the First UK COVID-19 Lockdown (Research Report No. 1). University of Surrey. https://openresearch.surrey.ac.uk/esploro/outputs/report/Gardens--Wellbeing-During-the-First/99587222502346.

[72] MacMillan Cancer Support (2020). The Forgotten ‘C’? The Impact of COVID-19 on Cancer Care. https://www.macmillan.org.uk/_images/forgotten-c-impact-of-covid-19-on-cancer-care_tcm9-359174.pdf.

[73] Cameron R.W., Blanuša T., Taylor J.E., Salisbury A., Halstead A.J., Henricot B., Thompson K. (2012). The domestic garden: Its contribution to urban green infrastructure. Urban For. Urban Green..

[74] Freeman C., Dickinson K.J., Porter S., van Heezik Y. (2012). “My garden is an expression of me”: Exploring householders’ relationships with their gardens. J. Environ. Psychol..

[75] Maat K., De Vries P. (2006). The influence of the residential environment on green-space travel: Testing the compensation hypothesis. Environ. Plan. A.

[76] Farahani L.M., Maller C., Phelan K. (2018). Private Gardens as Urban Greenspaces: Can They Compensate for Poor Greenspace Access in Lower Socioeconomic Neighbourhoods?. Landsc. Online.

[77] Rodiek S. (2014). Influence of an outdoor garden on mood and stress in older adults Influence of an Outdoor Garden on Mood and Stress in Older Persons. J. Ther. Hortic..

[78] Milligan C., Gatrell A., Bingley A. (2004). ‘Cultivating health’: Therapeutic landscapes and older people in northern England. Soc. Sci. Med..

[79] Goto S., Kamal N., Puzio H., Kobylarz F., Herrup K. (2014). Differential responses of individuals with late-stage dementia to two novel environments: A multimedia room and an interior garden. J. Alzheimer’s Dis..

[80] Goto S., Gianfagia T.J., Munafo J.P., Fujii E., Shen X., Sun M., Herrup K. (2017). The power of traditional design techniques: The effects of viewing a Japanese garden on individuals with cognitive impairment. Health Environ. Res. Des. J..

[81] Cervinka R., Schwab M., Schönbauer R., Hämmerle I., Pirgie L., Sudkamp J. (2016). My garden–my mate? Perceived restorativeness of private gardens and its predictors. Urban For. Urban Green..

[82] Stigsdotter U.A., Grahn P. (2004). A garden at your doorstep may reduce stress—Private gardens as restorative environments in the city. Proceedings of the Open Space-People Space International conference on Inclusive Outdoor Environments.

[83] Van Den Berg A.E., Custers M.H. (2011). Gardening promotes neuroendocrine and affective restoration from stress. J. Health Psychol..

[84] Young C., Hofmann M., Frey D., Moretti M., Bauer N. (2020). Landscape and Urban Planning Psychological restoration in urban gardens related to garden type, biodiversity and garden-related stress. Landsc. Urban Plan..

[85] Ekkel E.D., de Vries S. (2017). Nearby green space and human health: Evaluating accessibility metrics. Landsc. Urban Plan..

[86] Hand K.L., Freeman C., Seddon P.J., Recio M.R., Stein A., Van Heezik Y. (2017). The importance of urban gardens in supporting children’ s biophilia. Proc. Natl. Acad. Sci. USA.

[87] Fuller R.A., Irvine K.N., Devine-wright P., Warren P.H., Gaston K.J. (2007). Psychological benefits of greenspace increase with biodiversity. Biol. Lett..

[88] Venter Z.S., Barton D.N., Gundersen V., Figari H., Nowell M. (2020). Urban nature in a time of crisis: Recreational use of green space increases during the COVID-19 outbreak in Oslo, Norway. Environ. Res. Lett..

[89] Genter C., Roberts A., Richardson J., Sheaff M. (2015). The contribution of allotment gardening to health and wellbeing: A systematic review of the literature. Br. J. Occup. Ther..

[90] Gesler W. (1996). Lourdes: Healing in a place of pilgrimage. Health Place.

[91] English J., Wilson K., Keller-Olaman S. (2008). Health, healing and recovery: Therapeutic landscapes and the everyday lives of breast cancer survivors. Soc. Sci. Med..

[92] Bell S.L., Foley R., Houghton F., Maddrell A., Williams A.M. (2018). From therapeutic landscapes to healthy spaces, places and practices: A scoping review. Soc. Sci. Med..

[93] Coyle F. (2004). “Safe space” as counter-space: Women, environmental illness and “corporeal chaos”. Can. Geogr..

[94] Lewicka M. (2011). Place attachment: How far have we come in the last 40 years?. J. Environ. Psychol..

[95] Pouso S., Borja Á., Fleming L.E., Gómez-Baggethun E., Mathew P. (2021). Maintaining contact with blue-green spaces during the COVID-19 pandemic associated with positive mental health. Sci. Total Environ..

[96] Ottosson J., Grahn P. (2008). The role of natural settings in crisis rehabilitation: How does the level of crisis influence the response to experiences of nature with regard to measures of rehabilitation?. Landsc. Res..

[97] Wells N.M., Evans G.W. (2003). Nearby Nature: A Buffer of Life Stress Among Rural Children. Environ. Behav..

[98] Largo-Wight E., O’Hara B.K., Chen W.W. (2016). The efficacy of a brief nature sound intervention on muscle tension, pulse rate, and self-reported stress: Nature contact micro-break in an office or waiting room. Health Environ. Res. Des. J..

[99] Neill C., Gerard J., Arbuthnott K.D. (2019). Nature contact and mood benefits: Contact duration and mood type. J. Posit. Psychol..

[100] Galea S., Merchant R.M., Lurie N. (2020). The mental health consequences of COVID-19 and physical distancing: The need for prevention and early intervention. JAMA Intern. Med..

[101] Nisbet E.K., Zelenski J.M. (2011). Underestimating nearby nature: Affective forecasting errors obscure the happy path to sustainability. Psychol. Sci..

[102] Gammon S., Ramshaw G. (2021). Distancing from the Present: Nostalgia and Leisure in Lockdown. Leis. Sci..

[103] Riva G., Bernardelli L., Browning M.H.E.M., Castelnuovo G., Cavedoni S., Chirico A., Cipresso P., Maria D., De Paula B., Di Lernia D. (2020). COVID Feel Good—An Easy Self-Help Virtual Reality Protocol to Overcome the Psychological Burden of Coronavirus. Front. Psychiatry.

[104] White M.P., Yeo N.L., Vassiljev P., Lundstedt R., Wallergård M., Albin M., Lõhmus M. (2018). A prescription for “nature”—The potential of using virtual nature in therapeutics. Neuropsychiatr. Dis. Treat..

[105] Verderber S. (1986). Dimensions ofperson-window transactionsin the hospital environment. Environment and behavior. Environ. Behav..

[106] Musselwhite C. (2018). The importance of a room with a view for older people with limited mobility. Qual. Ageing Older Adults.

[107] Farley K.M., Veitch J.A. (2001). A Room with a View: A Review of the Effects of Windows on Work and Well-Being.

[108] Van Esch E., Minjock R., Colarelli S.M., Hirsch S. (2019). Office window views: View features trump nature in predicting employee O ffi ce window views: View features trump nature in predicting employee well-being. J. Environ. Psychol..

[109] Bartalucci C., Bellomini R., Luzzi S., Pulella P., Torelli G. (2021). A survey on the soundscape perception before and during the COVID-19 pandemic in Italy. Noise Mapp..

[110] Ferraro D.M., Miller Z.D., Ferguson L.A., Taff B.D., Barber J.R., Newman P., Francis C.D. (2020). The phantom chorus: Birdsong boosts human well-being in protected areas: Phantom chorus improves human well-being. Proc. R. Soc. B Biol. Sci..

[111] Ratcliffe E., Gatersleben B., Sowden P.T. (2013). Bird sounds and their contributions to perceived attention restoration and stress recovery. J. Environ. Psychol..

[112] Ratcliffe E., Gatersleben B., Sowden P.T. (2016). Associations with bird sounds: How do they relate to perceived restorative potential?. J. Environ. Psychol..

[113] Van Hedger S.C., Nusbaum H.C., Clohisy L., Jaeggi S.M., Buschkuehl M., Berman M.G. (2019). Of cricket chirps and car horns: The effect of nature sounds on cognitive performance. Psychon. Bull. Rev..

[114] Pizzoli S.F.M., Marzorati C., Mazzoni D., Pravettoni G. (2020). Web-based relaxation intervention for stress during social isolation: Randomized controlled trial. JMIR Ment. Health.

[115] Eadson W., Harris C., Parkes S., Speake B., Dobson J., Dempsey N. (2021). Why Should We Invest in Parks? Evidence from the Parks for People Programme; Forin People Programme. https://www.heritagefund.org.uk/sites/default/files/media/attachments/ParksforPeoplereport.pdf.

[116] Pritchard A., Richardson M., Sheffield D., McEwan K. (2020). The Relationship Between Nature Connectedness and Eudaimonic Well-Being: A Meta-analysis. J. Happiness Stud..

[117] Lumber R., Richardson M., Sheffield D. (2017). Beyond knowing nature: Contact, emotion, compassion, meaning, and beauty are pathways to nature connection. PLoS ONE.

[118] Coventry P.A., Brown J.V.E., Pervin J., Brabyn S., Pateman R., Breedvelt J., Gilbody S., Stancliffe R., McEachan R., White P.C.L. (2021). Nature-based outdoor activities for mental and physical health: Systematic review and meta-analysis. SSM Popul. Health.

[119] Robinson J., Breed M. (2019). Green Prescriptions and Their Co-Benefits: Integrative Strategies for Public and Environmental Health. Challenges.

[120] Chauvette A., Schick-Makaroff K., Molzahn A.E. (2019). Open data in qualitative research. Int. J. Qual. Methods.

