# Supplementary material for: Understanding the Role of Nature Engagement in Supporting Health and Wellbeing during COVID-19
Source: Int J Environ Res Public Health. 2022 Mar 25;19(7):3908. doi: 10.3390/ijerph19073908 (PMC8997429; doi:10.3390/ijerph19073908)
Supplement: Supplementary file 1 [file ijerph-19-03908-s001.zip › Figure S1. Illustration of results.pdf]

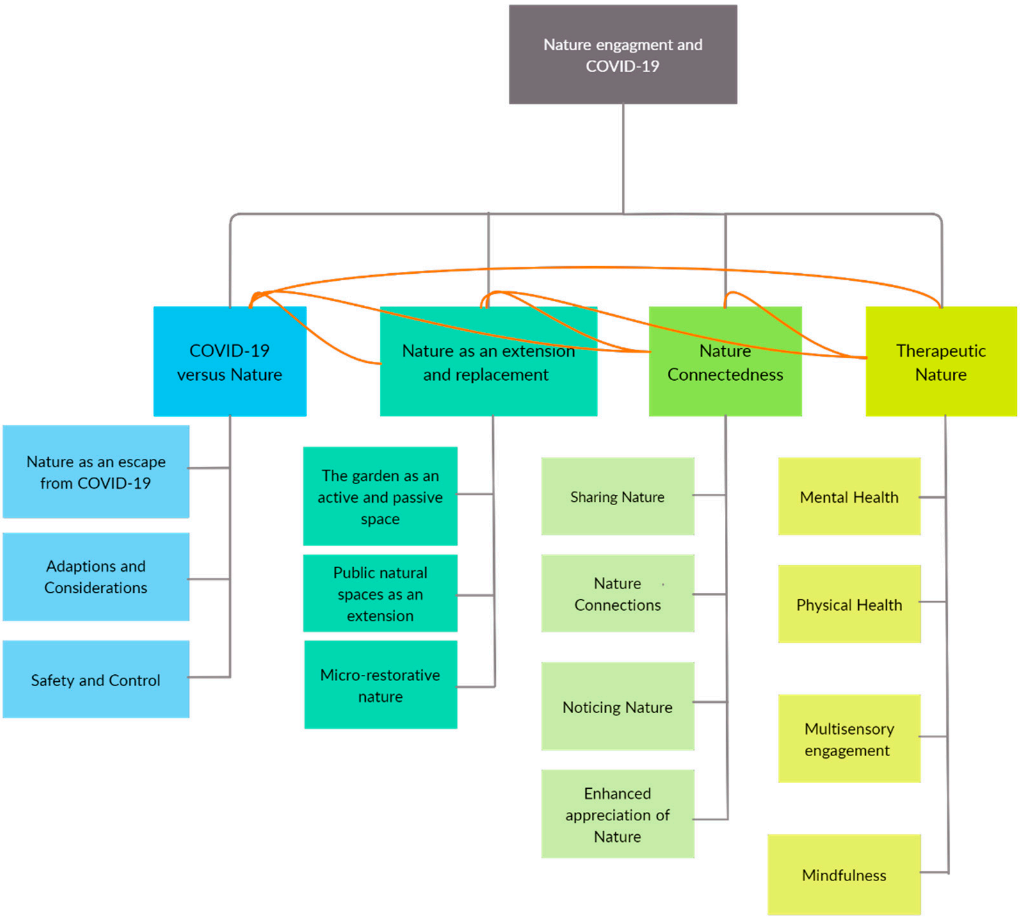

Figure S1. : Illustration of results.

Illustration of the relationships and links between superordinate themes and subthemes.
