# Supplementary material for: Understanding the Role of Nature Engagement in Supporting Health and Wellbeing during COVID-19
Source: Int J Environ Res Public Health. 2022 Mar 25;19(7):3908. doi: 10.3390/ijerph19073908 (PMC8997429; doi:10.3390/ijerph19073908)
Supplement: Supplementary file 1 [file ijerph-19-03908-s001.zip › Table S1 Interview Topic Guide.pdf]

Table S1: Interview Topic Guide

Public Natural Spaces

- Have you made use of or being able to make use of local natural environments during Covid-19? Why/Why not?
- Can you describe the types of natural environments you have used?
  - What were your reasons for using them? How did you use them? How was this different from how you would normally use these spaces?
  - What were the benefits to you in using them (i.e., Psychological; Social; Physical/General Health)?
  - Was there anything challenging for you in accessing or using local natural environments during Covid-19?
  - Was there any different benefits or challenges in using different types of natural environments (e.g., greenspaces (urban parks) compared to bluespaces (lakes, rivers))?

Private Natural spaces

- Do you have access to a private garden where you currently live?
  - Have you used this space during Covid-19? Why/Why not?
  - In what ways did you use this space? How was this different from how you would normally use this space?
  - What were the benefits to you in using this space (i.e., Psychological; Social; Physical/General Health)?
  - Was there anything you found challenging about using this/space?
  - How was this different from accessing/engaging with other types of natural environments?

Digital nature engagement

- Have you used digital technologies (i.e., social media, online games, multimedia and mobile phones) to engage with nature during the pandemic?
  - How was this different from what you would have done previously?
  - What were the benefits to you in using digital technologies to engage with nature (i.e., Psychological; Social; Physical/General Health)?
  - Was there anything you found challenging in accessing/using digital technologies?
  - How was your experience of engaging with nature digitally (e.g., online) compared to engaging with actual nature (e.g., nature outdoors or nature indoors)?

Other nature engagement

- Were there any other activities/ways you engaged with nature during the lockdown?
  - What were these activities? Why did you use these activities?
  - How was this different from what you would have done previously?
  - What were the benefits to you in engaging with nature in this way (i.e., Psychological; Physical/General Health)?
  - Was there anything you found challenging about engaging in this way?

Perceptions/Attitudes to Natural Environment Engagement

- Considering the types of natural environment engagement, you have identified, do you have a preferred environment or type of nature engagement you seek out to manage the current situation? Why?
- Have you engaged in any additional environmental-related activities during Covid-19 (for example, buying locally grown foods, recycling, buying eco-friendly products, activities that protect the environment)?

Classification: Restricted

    - How was this different from what you would have done previously?
    - What were the benefits to you in engaging in these activities (Psychological; Physical/General Health)?
    - Was there anything you found challenging about engaging in environmental-related activities during Covid-19?
    - Were there any alternative activities you engaged in as a response to not being able to do some of these activities?
- How do you think about the natural environments now compared to before Covid-19?
- How do you intend to use natural environments going forward?
