# Supplementary material for: Understanding the Role of Nature Engagement in Supporting Health and Wellbeing during COVID-19
Source: Int J Environ Res Public Health. 2022 Mar 25;19(7):3908. doi: 10.3390/ijerph19073908 (PMC8997429; doi:10.3390/ijerph19073908)
Supplement: Supplementary file 1 [file ijerph-19-03908-s001.zip › Table S2 Consolidated criteria for reporting qualitative studies (COREQ) 32-item checklist.pdf]

Table S2 Consolidated criteria for reporting qualitative studies (COREQ): 32-item checklist

Domain 1: Research team and reflexivity.

		**Personal Characteristics**	
1.	Interviewer/facilitator	Which author/s conducted the interview or focus group?	Pg. 6
2.	Credentials	What were the researcher's credentials?	Pg. 6
3.	Occupation	What was their occupation at the time of the study?	Pg. 6
4.	Gender	Was the researcher male or female?	Pg. 1
5.	Experience and training	What experience or training did the researcher have?	Pg. 6

		**Relationship established**	
6.	Relationship with participants	Was a relationship established prior to study commencement?	Pg. 3
7.	Participant knowledge of the interviewer	What did the participants know about the researcher? e.g. personal goals, reasons for doing the research	Pg. 4
8.	Interviewer characteristics	What characteristics were reported about the interviewer/facilitator? e.g. Bias, assumptions, reasons and interests in the research topic	Pg. 6

Domain 2: study design

		**Theoretical framework**	
9.	Methodological orientation and Theory	What methodological orientation was stated to underpin the study? e.g. grounded theory, discourse analysis, ethnography, phenomenology, content analysis	Pg. 3
		Participant selection	
10.	Sampling	How were participants selected? e.g. purposive, convenience, consecutive, snowball	Pg. 4
11.	Method of approach	How were participants approached? e.g. face-to-face, telephone, mail, email	Pg. 4
12.	Sample size	How many participants were in the study?	Pg. 4
13.	Non-participation	How many people refused to participate or dropped out? Reasons?	Pg. 4
		Setting	
14.	Setting of data collection	Where was the data collected? e.g. home, clinic, workplace	Pg. 4
15.	Presence of non-participants	Was anyone else present besides the participants and researchers?	Pg. 6
16.	Description of sample	What are the important characteristics of the sample? e.g. demographic data, date	Pg. 4-6

Classification: Restricted

17.	Interview guide	Were questions, prompts, guides provided by the authors? Was it pilot tested?	Supplementary file
18.	Repeat interviews	Were repeat interviews carried out? If yes, how many?	N/A
19.	Audio/visual recording	Did the research use audio or visual recording to collect the data?	Pg. 6
20.	Field notes	Were field notes made during and/or after the interview or focus group?	Pg. 6
21.	Duration	What was the duration of the interviews or focus group?	Pg. 6
22.	Data saturation	Was data saturation discussed?	N/A
23.	Transcripts returned	Were transcripts returned to participants for comment and/or correction?	No

Domain 3: analysis and findings

		**Data analysis**	
24.	Number of data coders	How many data coders coded the data?	Pg.6
25.	Description of the coding tree	Did authors provide a description of the coding tree?	Pg. 7
26.	Derivation of themes	Were themes identified in advance or derived from the data?	Pg. 6
27.	Software	What software, if applicable, was used to manage the data?	Pg. 6
28.	Participant checking	Did participants provide feedback on the findings?	No
		Reporting	
29.	Quotations presented	Were participant quotations presented to illustrate the themes / findings? Was each quotation identified? e.g., participant number	Pg. 7 - 20
30.	Data and findings consistent	Was there consistency between the data presented and the findings?	Pg. 7 - 20
31.	Clarity of major themes	Were major themes clearly presented in the findings?	Pg. 7 - 20
32.	Clarity of minor themes	Is there a description of diverse cases or discussion of minor themes?	Pg. 7 - 20

Classification: Restricted
